# A Qualitative Study of the Latter Effects of the COVID-19 Pandemic on Patients Living With Chronic Pain

**DOI:** 10.1177/23743735231199673

**Published:** 2023-09-13

**Authors:** Eleni Hapidou, Victoria Borg Debono, Saxon Schwarz, Jennifer Anthonypillai

**Affiliations:** 1 Department of Psychiatry and Behavioral Neurosciences, 3710 McMaster University, Hamilton, Canada; 2 Department of Health Research Methods, Evidence, and Impact, 3710 McMaster University, Hamilton, Canada; 3 Department of Psychology, Neuroscience and Behavior (PNB), 3710 McMaster University, Hamilton, Canada; 4 Michael G. DeGroote Pain Clinic, 3708 Hamilton Health Sciences, Hamilton, Canada

**Keywords:** COVID-19, patient perspectives/narratives, quality of life, qualitative methods, chronic pain

## Abstract

This qualitative study examined the effects of the COVID-19 pandemic on the lives of patients living with chronic pain (CP). Patients referred to an interdisciplinary pain management program between July and December of 2021 were asked to respond to the question: “How did the COVID-19 pandemic affect your life?” Fifty-four patients provided comments in response to this question. The comments were analyzed using an inductive approach. Ten themes emerged: (1) psychological state, (2) limitations on social life and activities, (3) minimal to no effect, (4) beliefs and opinions associated with COVID-19, (5) family dynamics, (6) healthcare disruptions, (7) pandemic-related fear, (8) changes in work, (9) change in pain, and (10) getting COVID-19. These themes mirror those found during the onset of the pandemic, with the addition of theme #4. Themes demonstrate the challenges experienced by individuals living with CP, in addition to new developments in the latter portion of the COVID-19 pandemic. It is important to understand the ramifications of shutdowns, so we are better able to address issues that occur in their aftermath.

## Introduction

### Covid-19 Shutdown's Impact on Access to Chronic Pain Treatment

The COVID-19 pandemic brought lockdowns, stay-at-home orders, physical distancing, and closure of nonessential services, all of which have been lifted at this time. These changes in daily life especially impacted people living with chronic pain (CP)^1^ who experienced barriers in receiving treatment for their pain and disruption to their psychological and physical pain treatment options.^2^ Closures of clinics and exercise facilities, and cancelations of appointments were key factors in reducing access to healthcare services.^3^ Closures resulted in a shift away from multidisciplinary pain treatment including psychological, physical, or complementary treatments in addition to pharmacological options.^2^ In response to reduced access to treatment, individuals with CP have had to adapt and modify their treatments and self-management strategies.^3^ Remote solutions such as telemedicine were more heavily utilized to maintain continuity of care and helped overcome geographical and disability barriers for those with CP.^4,5^ Where there have been deficits in the healthcare system, remote options have been used to bridge the gap in providing more well-rounded treatment approaches.^6^

People living with CP experienced significant psychological effects, with almost half of the respondents of the pan-Canadian study reporting moderate to severe distress because of the COVID-19 pandemic.^7^ Those who felt most vulnerable or were concerned for the health of others during the pandemic were most likely to report psychological challenges. Stress appraisal was most strongly associated with pain status and psychological distress, demonstrating that the effects on psychological well-being are implicated in pain worsening.^7^

Regarding daily functioning, those with CP experience a high degree of functional impairment. This is indicated through high levels of COVID-19-related fear and avoidance, depression, and general impairment of daily functioning, all factors associated with increased pain.^8^

### Veterans

Normally, veterans experience CP more frequently than nonveterans, and are, therefore, just as susceptible to COVID-19-related changes as nonveterans.^9^ A significant number of Veterans living with CP have experienced worsened pain since the beginning of the pandemic, moderate to severe psychological distress, and have had to alter their pain treatments in response to the pandemic just like nonveterans.^9^ The Intensive Chronic Pain Management Program at the Michael G. DeGroote Pain Clinic treats Veterans so we also included them in this study to better understand and detect if there were any differences in how the COVID-19 pandemic affected their lives compared to nonveterans to ensure we were capturing this population.

### Significance of This Research

The purpose of this qualitative study is to explore the psychosocial effects of the COVID-19 pandemic on a sample of veterans and civilians with CP. This study contributes to the literature by exploring how patients with CP experienced the effects of the pandemic over the long term. It is also important to understand the ramifications of the COVID-19 shutdowns, so we are better able to anticipate and address the issues they cause in their aftermath and be in a better position to develop preventative measures to adequately help people living with CP with future health crises.

### Study Context

The Intensive Program is a 5-week pain management program offered to patients with CP at the Michael G. DeGroote Pain Clinic. Prior to the pandemic, the program was conducted in-person only. A virtual program was developed to facilitate conducting the program while the COVID-19 restrictions were in place where possible. The patient population is half civilians and half veterans at any given time. This study took place during the Delta outbreak.

## Methods

### Ethics

This study protocol received approval by the Hamilton Integrated Research Ethics Board (HiREB) on August 13, 2021. The HiREB project # is 11166. Data were collected as per HiREB policy and confidentiality was maintained.

### Recruitment

Between July 2021 and December 2021, patients who were considered new intake and partook in an initial psychological assessment were included in this study. Patient participant identification and their responses were anonymized.

### Study Setting

The participants were being interviewed as part of an interdisciplinary assessment to determine eligibility for recommendation to attend the Intensive Chronic Pain Management Program at the Michael G. DeGroote Pain Clinic in Hamilton, ON, Canada. At the time, due to the pandemic, about half of these assessments were conducted virtually as per the patients’ preference.

### Data Collection

For this study, patients were interviewed by a registered psychologist who conducted psychodiagnostic interviews with patients with lived experiences related to CP during the COVID-19 era. Comments for this study were collected between July 2021 to December 2021 as part of a larger interdisciplinary assessment to determine patients’ eligibility to attend the Intensive Chronic Pain Management Program at the Michael G. DeGroote Pain Clinic, Hamilton, ON, Canada. Patients consented to their assessment ahead of time. As part of the psychological assessment, patients were asked, “How did the COVID-19 pandemic affect your life?” halfway through the assessment interview. This was the only framing question for this study. Participants were also encouraged not to answer any questions they did not feel comfortable answering during the assessment interview. The psychologist at this time took interview notes and those notes were later anonymized to create textual data for analysis in REDCap. Patients’ comments were anonymized for blind review and analysis by other researchers.

Data were collected, managed, and secured in REDCap hosted on McMaster University servers. REDCap is a secure, web-based software platform for data collection. Surveys were saved in REDCap and de-identified using REDCap IDs. Patient names were removed from REDCap and stored in a password-protected Excel® only accessible by the research coordinator.

### Data Analysis

Thematic analysis of comments was conducted using a qualitative descriptive phenomenological approach that allows for inductive thematic analysis. Thematic analysis used in our study was applied to a set of interview transcripts and/or notes made by the interviewing psychologist. The researchers closely examined the data to identify common themes—topics, ideas, and patterns of meaning that come up repeatedly. The process of thematic analysis occurred inductively meaning that the themes identified are strongly linked to the data and that the process of coding occurs without trying to fit the data into a preexisting theory or framework.^10^

The process used for conducting a thematic analysis based on a descriptive phenomenological approach involved us going from the original data (interview transcripts or notes) to identifying meanings, organizing these meanings into patterns, and then writing the results as themes related to the study aim and context. When reporting the findings, the themes were described first, along with descriptive text and quotes to illustrate them. This resulted in a meaningful text that organizes the meanings found from participants’ experiences into themes. This was done independently and concurrently by 3 researchers using Microsoft Excel®. Then all 3 researchers met with the research lead to discuss their codes, ensure agreement, and solidify themes. Through sampling saturation, anonymization, and corroboration, the quality, credibility, and integrity of data analysis were ensured.

## Results

### Demographics

Comments were collected from 54 patients with CP, 23 Veterans, and 31 civilians, 55.5% male, 61.8% married/common-law, 21 to 79 years old with an average (SD) of 49 years (12.7). CP was 48.1% from a motor vehicle accident, 40.7% from a work/service-related injury, 5.5% from neither and 1.8% from both. Patients had been experiencing pain for an average (SD) of 11 years (11).

### Qualitative Analysis

Fifty-four patients provided comments in response to the prompt: “How did the COVID-19 pandemic affect your life?” Analysis of the comments led to 10 themes (Table 1).

**Table 1.** Themes, Number of and Supporting Comments.

Theme	No of Comments	Example of comment supporting theme
1. Psychological state	34	**Increased frustration, anxiety, and worsening of pre-COVID-19 challenges:** A male civilian commented, “I was already eight feet under due to the car accident. It affected me more. It's a disease out there. Another problem that causes me more anxiety and restrictions.” **Decreased anxiety, benefit from staying in and avoiding crowds:** A male veteran reported, “It made it easier. Less people, less congestion, less panic attacks.”
2. Limitations on social life and activities	17	**Isolation and limitation on everyday activities:** A female veteran reported, the pandemic affected her hard, “being confined at home, couldn’t see people, couldn’t visit people, couldn’t go shopping.” **Disruption of leisure activities:** A male civilian commented, “We don’t go out to dinners as much as we had. Just started going to breakfast. Changed our lifestyle. Canceled three cruises.”
3. Minimal to no effect	16	A female civilian reported, “hasn’t affected me at all; still doing the same things I was doing beforehand.”
4. Beliefs and opinions associated with COVID-19	16	**Antimasking belief and COVID-19 denial:** A male veteran reported, it “angers” him to wear a mask he does not need to wear. The COVID pandemic has affected him “a lot. I was trained in nuclear and biological warfare. You cannot catch it outside. Some people are brainwashed, and this has affected me.” **Opinions supporting guidelines:** A female civilian reported experiencing strong reactions to people not following rules, like wearing a mask when it is not something she would have noticed before. **Challenges following guidelines:** A female civilian reported, “It has cost me further delays due to cognitive issues, trying to navigate, follow signs and arrows … difficult and overwhelming to take on the rules and information.” **Positive opinion of vaccination:** A male civilian indicated he is “twice vaccinated” and feels “hopeful.”
5. Family dynamics	11	**Conflict due to isolation:** A female veteran reported that COVID put a wedge between her and her children as they were locked in at home together.
6. Healthcare disruptions	10	**Therapies for mental health put on hold:** A male veteran commented, “Difficult to have the supports I need”; he has not seen his psychologist or psychiatrist in one and a half years.
7. Pandemic-related fear	7	**Fear of the COVID-19 variants:** A female veteran reported she was “still scared to death because of the variants.”
8. Changes in work	4	**Loss of employment:** A female civilian commented, “I got laid off due to COVID just like half of the world.”
9. Change in pain	4	**Increased pain following COVID-19 infection:** A female veteran reported that, since she had a COVID infection, her pain has increased quite dramatically.
10. Getting COVID-19	3	**Caught COVID-19** A female veteran caught COVID in January 2020 before it was announced, from a student at school. She had all the symptoms, lost taste for six months, and had a fever.

(1) Changes in psychological state

Thirty-four comments contributed to this theme describing changes in psychological state, separated into 2 subthemes: negative or positive change. Negative change (22 comments) includes reports of increased frustration, anxiety, and worsening of pre-COVID-19 challenges. Positive change (12 comments) includes reports that staying home and avoiding social activities and interactions had provided relief.

(2) Limitations on social life and activities

Seventeen comments contributed to this theme describing how the lockdowns and guidelines limited the ability to be social and engage in everyday activities. Most patients reported feelings of isolation and challenges associated with not seeing friends and family, and limitations on recreational activities such as going out to dinner, traveling, and going to the gym. Most patients reported a change in lifestyle.

(3) Minimal to no effect

Sixteen comments contributed to this theme. Many patients reported the pandemic had minimally affected their life; they had not been going out previously due to CP or are still doing the same things they had been doing before the pandemic.

(4) Beliefs and opinions associated with COVID-19

Sixteen comments contributed to this theme describing patients’ reported beliefs and opinions associated with COVID-19, broken down into 3 subthemes: (a) beliefs about the COVID-19 virus and pandemic, (b) opinions on guidelines, and (c) opinions on vaccination. Under (a), some patients reported beliefs describing COVID-19 denial, or antimasking. Under (b), some patients reported frustration with guidelines and difficulty following them, while others reported that they respected or followed guidelines with ease. Under (c), many patients reported already being vaccinated, 1 patient reported vaccination hesitancy, and 1 patient reported concern about others around him not being vaccinated.

(5) Family Dynamics

Eleven patients contributed to this theme, describing how connections with family have been affected. Some patients reported that the pandemic had brought on conflict because of isolating together, while others reported it had brought them closer. Some patients reported experiencing greater support from family at home. One patient reported challenges associated with teaching kids at home due to school closures.

(6) Healthcare Disruptions

Ten comments contributed to this theme, which describes disruptions in accessing healthcare. Most patients reported complete interruptions in therapies and medical appointments as well as difficulty finding a doctor. Therapies put on hold included physiotherapy, occupational therapy, psychology, and psychiatry.

(7) COVID-19-related fear

Seven comments contributed to this theme, describing feelings of fear in relation to the pandemic. Patients reported fearing being in public, contracting COVID-19 with a pain condition, bringing home COVID-19 to elderly parents, and COVID-19 variants.

(8) Changes in work

Four comments contributed to this theme, describing changes in the employment of patients and their families due to the pandemic. Patients reported experiencing loss of employment due to COVID-19, while others and their families returned to work or worked from home. One patient reported starting to work from home as a veteran, which eased the transition from military to civilian life.

(9) Change in pain

Four comments contributed to this theme. Patients reported an increase in pain due to masking, as well as since being infected with COVID-19.

(10) Getting COVID-19

Three comments contributed to this theme. Two patients were infected themselves, while 1 patient's family was infected.

### Overall Findings Based on Themes

The most represented theme was “changes to psychological state,” revealing a profound and varied psychological impact of the pandemic on individuals with CP, including increased anxiety, frustration, and isolation-related challenges. Some patients, however, reported positive psychological outcomes, experiencing reduced stress and an overall improvement in their quality of life. “Limitations on social life and activities” due to public health policies were common, affecting day-to-day activities and lifestyle changes, leading to psychological distress. A notable theme emerged concerning “beliefs and opinions associated with COVID-19,” showing diverse views on the virus, guidelines, and vaccination. Healthcare disruptions were also evident, impacting pain management and causing emotional distress. Despite the pandemic's later stages, “pandemic-related fear” persisted, particularly for those with CP, underlining the need for support. Few patients reported changes in employment, and only a few experienced an increase in pain due to COVID-19.

## Discussion

This study explores the effects of the latter portion of the COVID-19 pandemic on 54 individuals with CP, assessed at the Intensive Chronic Pain Management Program at the Michael G. DeGroote Pain Clinic over a 6-month period in 2021. Patients answered a question on how the COVID-19 pandemic had affected their lives. Ten themes emerged following analysis of these answers: (1) Changes to psychological state, (2) limitations on social life and activities, (3) minimal to no effect, (4) beliefs and opinions associated with COVID-19, (5) family dynamics, (6) healthcare disruptions, (7) pandemic-related fear, (8) changes in work, (9) change in pain, and (10) getting COVID-19.

The theme most represented across comments, “changes to psychological state,” demonstrates how the COVID-19 pandemic has had a profound and varied psychological effect on individuals experiencing CP. This effect on mental health has been explored in the literature showing that individuals with CP are increasingly vulnerable to psychological distress in response to the pandemic.^7,9,11,12^ Patients whose answers referred to a negative psychological state reported increased anxiety, frustration, and an exacerbation of pre-COVID-19 challenges. Many patients reported psychological challenges associated with feelings of isolation, which is not surprising given that by the time of assessment, patients had endured lockdowns in the long-term.

Other patients reported a positive psychological state in response to the pandemic: a reduction in stress and anxiety, and an overall improvement in quality of life. Following public health guidelines, individuals spent more time resting, acceptably engaged less with others, and no longer missing out on activities they could not participate in before the pandemic. This effect demonstrates the variation in lived experiences of those with CP, as the pandemic has led to both significant psychological challenges and relief. The role of lived experience is, therefore, paramount when discussing the effects of COVID-19 on individuals with CP.

A theme that emerged from patients’ comments involves “limitations on social life and activities” due to public health policies and guidelines. Many patients reported feelings of isolation, with a significant impact on day-to-day and leisure activities and an overall change in lifestyle. These changes contribute to the psychological distress experienced by individuals with CP.^12^

In contrast to the previous theme, approximately the same number of patients reported that the pandemic had “minimal to no effect” on their lives. Patients reported no differences from before the onset of the pandemic to the date of assessment, often because they were already limited in their ability to leave home or socialize. This theme demonstrates the need for more research on ways to improve the quality of life of those with CP postpandemic.

Patients’ experiences varied in the theme of “family dynamics,” as some reported positive experiences of becoming closer to family and having increased social support. Others reported challenges with teaching children at home and conflict among family members. Patients also reported concern for the health of family members, which increases their vulnerability to psychological challenges.^7^ Future research should examine the impact of positive and negative family dynamics on health outcomes.

An interesting theme describes patients’ “beliefs and opinions associated with COVID-19.” Patients discussed the legitimacy and transmissibility of the virus itself, as well as beliefs related to the sociocultural contexts of the pandemic. Patients reported opinions on guidelines such as shutdowns and masking; some found no difficulty in following guidelines while others experienced increased delays, frustration, confusion, and overwhelm. Patients often reported the number of times they had been vaccinated, though some reported vaccination hesitancy, while others were concerned that others around them might not be vaccinated. Overall, the theme of COVID-19-related beliefs and opinions demonstrates that the pandemic has significant sociocultural implications.

A crucial theme describes “healthcare disruptions” caused by the pandemic. Patients reported interruptions of in-person healthcare services and treatments such as physiotherapy, occupational therapy, psychology, psychiatry, and family doctor's appointments. These interruptions have been widespread and have caused a shift away from multidisciplinary approaches to pain management.^4^ Loss of healthcare services has been shown to predict emotional distress.^12^ This is critical for those suffering from CP, as patients have been experiencing long-term interruptions in healthcare services.

While patients had been able to surpass the initial shock of the pandemic during the time of assessment, “pandemic-related fear” was still described in the comments. Patients reported fear of being in public, and of catching COVID-19, especially with having a chronic illness. Patients also reported fear of the COVID-19 variants. Thus, individuals with CP have remained fearful into the latter portion of the pandemic. This is unsurprising given that individuals with CP experience increased pandemic-related worry and fear compared to those without CP.^11^ This theme highlights the additional emotional and psychological challenges associated with experiencing CP during a pandemic and demonstrates a considerable need for support.

While few patients reported a change in employment, such a change is notably significant for those experiencing a transition. Patients reported a loss of employment, transitioning to working from home, rejoining the army, and transitioning from military to civilian life. Changes in employment in this population have been demonstrated to be stressors, especially when individuals are living at the intersection of CP and low socioeconomic status.^13^

Interestingly, only 4 patients reported their pain, with only 2 reporting an increase. Patients reported an increase in pain following a COVID-19 infection, and due to masking. While other causes were not reported, individuals with CP are susceptible to experiencing increased pain due to a variety of stressors and changes brought on by the pandemic, such as emotional distress and changes in routine.^12^

The final theme involved contracting the COVID-19 virus. Patients reported themselves and family members having the virus. Only 3 patients’ comments contributed to this theme which demonstrates the isolation of these patients given that so few contracted the virus later in the pandemic.

The COVID-19 pandemic has not affected everyone equally, with vulnerable groups more heavily impacted.^1^ This study explores the most salient themes relevant to the effects of the pandemic on individuals with CP. There was no clear preponderance of any theme represented by a particular group, as themes were distributed evenly among the sample. While the short-term impact of the early pandemic has been studied with reference to pain intensity, pain severity, depression, anxiety, and pain-related challenges,^14,15^ the longer-term effects of the pandemic have not yet been explored. Future research may examine the long-term repercussions of the pandemic on the CP population.

A study by Mohamed Ali *et al*^16^ posed the same question to the same demographic at the onset of the pandemic, June 2020–June 2021. The findings of this study mirror those found by Mohamed Ali *et al* with the addition of theme #4 on “beliefs and opinions associated with COVID-19.” All other themes seem to have persisted in the latter portion of the pandemic. Noteworthy in theme consistency was the theme “minimal to no effect on life.” This reflects the deep impact that CP already has on the quality of life in people living with CP.

Other studies have also reported indirect effects of the pandemic and its impact on social life and activities, and access to health services. A conceptual model has been shared to explain the impact of COVID-19-related changes in the social environment on the health of patients with CP. The pandemic is seen as a social threat, compounding challenges that individuals with CP already face in maintaining social well-being. Measures such as lockdowns and social distancing exacerbate social isolation, increase family care responsibilities, cause interpersonal conflicts, limit access to pain care, and foster perceptions of societal injustice and inequality. Cumulative evidence supports this model, showing that these social threats worsen pain levels, pain-related symptoms, psychosocial function, and pain interference.^17-22^ This evidence was observed in a cross-sectional online survey of community-dwelling adults with CP, who reported increased average pain intensity, pain flare-up episodes, distress, pain interference, and impaired sleep during the pandemic.^23^

However, a study by Ziadni *et al* found that the pandemic did not adversely affect physical and mental health among patients seeking treatment for pain at the Stanford Pain Center.^24^ Their post-COVID-19 cohort reported small but significant increases in emotional support and no significant changes in social isolation and social satisfaction. Even though this is contrary to other recently published findings,^11,12^ an important distinction is that the current study sample was in treatment at the Stanford Pain Center and continued to receive intense virtual treatment during the pandemic. The conflicting evidence here suggests that when there are certain societal and major health care supports in place, like continuous and intensive virtual access to care and treatment, this can ameliorate the negative effects the pandemic had on CP and life overall.

While our current study provides a snapshot of the early and latter effects of the pandemic, future research may include comparisons of themes across the pandemic timeline as well as an exploration of how themes are related and whether certain social supports in place both at home and on a larger societal level alleviate some of the negative effects/themes the pandemic had on life.

## Conclusion

This qualitative study contributes to the literature exploring the psychosocial impacts of the COVID-19 pandemic on individuals with CP. Findings demonstrate changes in psychological well-being, disruptions in access to healthcare, limitations in social interaction and recreational activities, beliefs and opinions associated with COVID-19, and minimal to no effect on life, to be the most salient themes. Of particular interest was the theme “minimal to no effect on life” which highlights the impact CP has on one's quality of life and in that it can equate to a global pandemic's impact on society. More research needs to be done to investigate how quality of life can improve in those living with CP.
